# “I Don’t Understand!”: Toward a Model to Evaluate the Role of User Story Quality

**DOI:** 10.1007/978-3-030-49392-9_7

**Published:** 2020-05-06

**Authors:** Daniel Hallmann

**Affiliations:** 6 University of Oslo, Oslo, Norway; 7 Monash University, Clayton, VIC Australia; 8 IT University of Copenhagen, Copenhagen, Denmark; 9 University of British Columbia, Vancouver, BC Canada; 10 University of Bamberg, Bamberg, Germany; 11 University of Applied Sciences Dresden, Dresden, Germany

**Keywords:** Agile software development, User story quality, Developer experience, Shared mental model, Project success

## Abstract

User stories are popular for conveying requirements in agile software projects. Despite existing quality criteria, authors make formal mistakes that result in “bad” user story quality. If developers have insufficient experience in balancing quality problems, the creation of a shared mental model is impossible, thus increasing the risk of impacts on the project’s success. This article provides a work-in-progress research model to set these variables in relation and establish a systematic method to uncover answers regarding their correlation. Details on the effects support research in agile requirements engineering to gain a better understanding of cognitive processes in the comprehension of user stories. In addition, insights can help to develop design recommendations and AI tools to improve user stories. A first evaluation of the model provides promising insights into the behavior and forms a basis for future research.

## Introduction

In agile software projects, user stories are widely used to communicate requirements between authors such as a product owner—a business role in Scrum [4]—and developers. The short text documents specify a requirement in the form [title]—As [persona], I want [what] because [why]—[acceptance criteria]—[attachments] [4]. CCC (Card, Conversation, Confirmation) [10], INVEST (Independent, Negotiable, Valuable, Estimable, Small, Testable) [18] and Cohn’s Guidelines [4] are quality criteria, but mistakes from authors pervade, which can result in “bad” user stories. These might be incorrect or missing form fields (e.g., [acceptance criteria]) [4]. Additionally, developers with different experience levels work together on projects according to their career and time in a team [2].

Developers attempt to build a mental model [5] of the implementation steps and necessary effort based on problems in user stories during an estimation session, such as planning poker [18]. Without sufficient knowledge to balance inadequate information, it is not surprising that developers become frustrated and respond with “*I don’t understand!*”. These individual problems can prevent the forming of a shared mental model [5] between the author and developers and increase the risk of impacts on project success, such as lengthy discussions or unnecessary work [2].

Empirical work provides analyses of user stories in estimation sessions to identify important factors for reasonable estimates of story sizes [9, 12]. Accordingly, groups generate better results than do single individuals, and sufficient experience is essential for estimating coarse-grained user stories. Within the empirical studies, the focus is on optimizing the estimates made by experts. However, the existing research lacks a cognitive psychological perspective on understanding the content—especially in the case of issues in user story quality—as a further factor for obtaining proper estimates. For example, it is currently unclear how variances in user story quality with varying levels of developer experience affect the understanding or shared mental model [5] between the author and developers. If there is no shared view, the question of the impact on project success arises, a research gap we address in this paper. As a starting point for our research, we therefore ask the following research question:

**RQ:***What is the relationship between user story quality, developer experience, shared mental model between the author and developers, and project success?*

If we know details of the relationship, we can make statements regarding the effect of user story quality—and their “bad” and “good” variations—on human and project factors. We can close the gaps in agile requirements engineering research, especially in the introduction of user story comprehension as a level for evaluating the correctness of estimates. Subsequently, if we know the “bad” aspects, we can then create “good” aspects in design recommendations, thus helping authors in practice when they write user stories. In addition, it is conceivable to provide an intelligent AI tool support for the creation of user stories.

In this paper, we present a model to systematically create and evaluate the relationship. The structure of the model is based on the cognitive psychological perspective, which allows for the definition of latent constructs and their theoretical relationship. In addition, the paper presents our multi-method approach, which addresses the empirical data collection and evaluation of the model. The latent constructs are not directly measurable, so the methodological section includes a presentation of the measurable indicators. The initial evaluation results of the model subarea for user story quality appear promising. The structure of indicators can represent the structure in our data set, which allows for first steps in determining the correlation to the other constructs. However, the instrument is not currently error free, and the measurements can be inaccurate. The first analysis suggests that the low data size and differences in the indicator variances may cause the weakness. In the subsequent steps of the evaluation, we analyze the causes in greater detail to correct the inaccuracies. For this purpose, we will extend the test with additional user stories from other completed projects.

We subsequently present our model in Sect. 2, which describes the constructs and a priori assumptions of the relationship. Then, Sect. 3 provides details of the methods and indicators with which we organize our collection and analysis of the empirical data. Finally, in Sect. 4 we present our initial results of the preliminary evaluation, conclusions, and upcoming steps.

## Research Model

We selected structural equation modeling [3] as the first approach to answer the research question and systematically determine the strength of the correlations between the research objects. This method is a well-known and widely used in psychology [3], social sciences [11], and information systems [19].

**Fig. 1.**
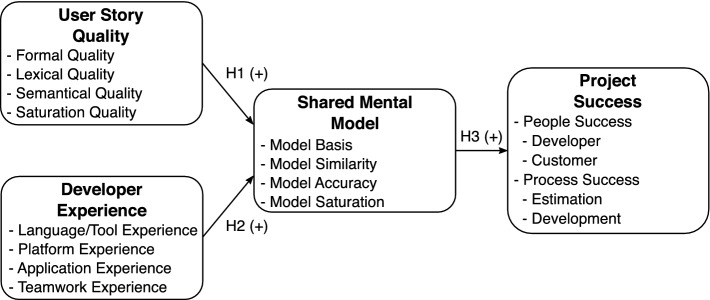
Research model

First, we extracted the four latent constructs user story quality, developer experience, shared mental model, and project success from the research question and built connections with hypotheses to form the basic structure. Each construct was then operationalized via four indicators to enable empirical data collection and evaluation. Figure 1 describes our model with constructs, hypotheses, and indicators. The following section presents details for the constructs and hypotheses. Details to the indicators are shown in the method Sect. 3.

**User Story Quality. ** The quality aspect of a user story is a central construct given its essential role in the problem statement. It represents the goodness and badness of the content and structure, with a focus on pure text features. Sentences, words, and syllables provide the basis for formal and semantical dimensions of quality. The number of filled-in form fields and business domain keywords can help to determine the degree of quality. Our approach reflects the existing quality criteria from CCC, INVEST, and Cohn’s Guidelines, especially regarding value generation and testability.

**Shared Mental Model. ** This construct describes the knowledge structures of the team members regarding the actual experiences and abilities of the team [5]. This knowledge creates the prerequisite for the coordinated joint processing of a task in a particular situation. Agile methodology focuses on individuals, interactions, and collaboration [1] in addition to a joint understanding of user stories in teams [4]. This orientation on humans and coordinated teamwork helps in accomplishing goals quickly.

A user story should be negotiable [18] to support conversation [10] and make estimation possible [18]. The format and quality criteria of the user story addresses text and in-person interaction. It should support information extraction and communication to form an individual and then team approach [4]. Therefore, a “good” user story with high readability and updates can support individual comprehension and a discussion promoting a shared mental model. Thus, we posit the following:

**H1:***The more that user story quality increases, the more shared is the mental model of the team members.*

**Developer Experience. ** Project insights are significant for development [2], especially in agile projects, with a focus on individuals [1]. Developers should understand technical (e.g., programming languages, tools), business domain (e.g., applications, requirements), and team (e.g., skills, attitudes) properties to make the proper decisions during implementation. Details of existing application components (e.g., sign in, security) are mandatory to determine which must be involved in implementation ideas. If the existing source code cannot be reused, everyone should be aware of technologies to generate a new approach.

Studies of shared mental models [5] reveal that personal properties, such as experience, impact the creation of a proper shared understanding in the team. In addition, results in software teams [2] identify the importance of prior familiarity with application components to obtain a stronger shared team and task mental model. Simultaneously, agile approaches mention the importance of having sufficient technical and domain knowledge in estimation sessions to arrive at a group agreement on a user story estimate [15]. We therefore propose the following:

**H2:***The more that developer experience increases, the more shared is the mental model of the team members.*

**Project Success.** The portion of the model that concerns project success covers economic aspects, with a focus on time and money [2]. It addresses the enhancements of projects in the light of people and process aspects. Therefore, developer experiences and requirements can positively influence the economic factors of a project. Triggers include sufficient developer experience and the fulfillment of requirements, such as user stories. All these aspects could save resources and must be supported.

Achieving a rapid understanding of a user story between all developers and a shared mental model with the author can support the motivation of developers and quick discussions in an estimation session. In addition, a shared understanding can increase appropriate implementation results later in a feature presentation and the satisfaction of an author and customer. The accepted feature without additional time for bug fixing also enables the direct development of additional software functions, which can have a positive effect on the performance and project schedule. Therefore, we posit the following:

**H3:***The more shared the mental model of the team members, the more supported is the project success.*

## Method

The methodological process to test the hypothesis and answer the research question begins with an ex-post analysis based on data from completed projects. It is common practice to conduct a preliminary study to gather initial findings, thus allowing for early model optimization [3]. Through exploratory factor analysis [3], we verify the consistency of indicators from subareas of the model. We began with existing user stories to evaluate the specification of user story quality, and our initial results are presented in Sect. 4. In addition, further improvements of indicators and an evaluation of the hypotheses are planned in two consecutive steps. First, we will perform a field study to gather new data from ongoing projects; this step is essential to address the data collection for all indicators, which allows for testing the entire model and our hypothesis. Afterward, we conduct an experiment based on small student teams to obtain data in a controlled situation. The focus here is on the evaluation of corner cases to stress the model and consolidate the parameters and predictions. User story quality is measured via document analysis and shared mental model and project success through observing estimation sessions. Project success is captured through document analysis and observation during the development phase and customer review. Obtaining data for developer experience is planned as a two-step process. Developers first rank their experience in a team meeting, and we then measure experience by observing estimation sessions.

The indicators for measuring the constructs are created based on conference feedback [7], a literature review [8], two expert interview studies, and research group meetings. The focus during creation was on proper content saturation, test quality criteria, linear behavior, and the reuse of project indicators [3]. In addition, a simple structure and fast value collection are aspects of indicator design. The scale of all indicators is positive with low (−) and high ($+$) values that define the low and high representation of the constructs. Details of each indicator and their specific scale and range are provided in the following section.

**User Story Quality.** *Formal quality* consists of the number of filled-in form fields (e.g., [title]) needed to identify a fulfillment status based on the story format [4]. A story should contain a set of information to maintain the promise for conversation. The scale is ordinal, with values of $0,1,\dots ,6$. *Lexical quality* is based on text properties, such as sentences and words, to compute the readability as a number [6]. It addresses the complexity of the lexical structure in which information is encoded that must be decoded by developers. The scale is rational, ranging from 0 to 100. Next, *semantical quality* measures the percentage of business keywords (e.g., VAT) versus the total number of words to indicate the strength of the value focus for the customer [18]. It highlights semantic details that developers must decipher to identify the concepts. The scale is rational, ranging from $0\%$ to $100\%$. Finally, *saturation quality* focuses on the number of changes in form fields (e.g., [what]) prior to implementation. It covers a saturation status because documents must be refined to increase their benefit as an information source [4]. The scale is rational, ranging from 0 to greater than 20.

**Developer Experience.** *Language and tool experience* addresses the average language (e.g., Java) and tool (e.g., editors) experience in time of the team [2]. Developers must be familiar with feature sets and limitations to design feasible implementation approaches. The scale is rational, ranging from 0 to greater than 6 years. *Platform experience* focuses on the infrastructure (e.g., database) experience necessary to manage components essential for the application to run [2]. The scale is rational, ranging from 0 to greater than 6 years. Next, *application experience* covers knowledge of the application components involved in the estimation process [2]. The team must implement the story content in the existing source code, and therefore, changes must be evaluated to provide a correct estimation. The scale is rational, ranging from 0 to greater than 6 years. Finally, *teamwork experience* covers the time that colleagues have worked in teams to gain thorough collaboration and communication social experience [1]. The scale is rational, ranging from 0 to greater than 6 years.

**Shared Mental Model.** *Model basis* describes the percentage of developers in the estimation session versus the total number of people on the team. A shared model is possible when many developers are part of a user story discussion to acquire similar information. The scale is rational, ranging from $0\%$ to $100\%$. *Model similarity* represents the percentage of developers versus the total number of people in the session, in which the estimate is equal to the final estimation result. The indicator adapts approaches from a similarity rating [13] to evaluate the team agreement to the story point value at the end of an estimation. The scale is rational, ranging from $0\%$ to $100\%$. Next, *model accuracy* focuses on the number of form fields mentioned during the process. It measures the model accuracy by providing the story details of the author to the developers. The scale is ordinal, with values of $0,1,\dots ,6$. Finally, *model saturation* [15] measures the number of questions asked by developers while discussing a story. Additional questions and answers can be helpful in refining ideas within the team. The scale is rational, ranging from 0 to greater than 20.

**Project Success.** *People success (developer)* is defined to measure the percentage of developers versus the total number of people in the estimation who are indicating happiness (e.g., through utterances) [17]. It addresses developers who can build a mental model [5]. The scale is rational, ranging from $0\%$ to $100\%$. *People success (customer)* covers the percentage of accepted acceptance criteria versus the total number of criteria in a customer review. It reflects a satisfaction status [14] and includes the quality criteria that stories should be testable. The scale is rational, ranging from $0\%$ to $100\%$. Next, *process success (estimation)* is the percentage of time that an estimation is lower than the highest duration for a story point value. It corresponds to quick discussions [15] and reflects quality criteria, in which stories should be negotiable. The scale is rational, ranging from $0\%$ to $100\%$. Finally, *process success (development)* measures the percentage of time that the team spends less on the implementation than the highest amount for that story size. Time is calculated for the initial coding, functional issues and bugs [16]. The scale is rational, with a range from $0\%$ to $100\%$.

## Preliminary Evaluation and Conclusions

As we worked on this paper, we began with the ex-post analysis and conducted an exploratory factor analysis [3] of the model subarea for user story quality. Our data set consisted of 74 user stories from a completed agile software development project. The project from the German automotive sector was conducted from March 2013 to December 2015 with a Scrum team of eight developers, one product owner, and one Scrum master. We considered only fully developed user stories that progressed equally through all development steps. Thus, the user stories had the same prerequisites of increasing comparability.

**Fig. 2.**
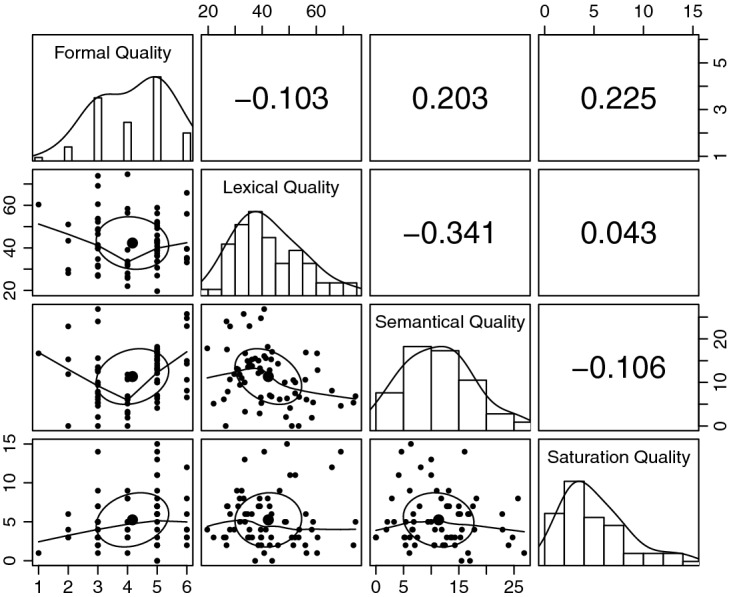
Distributions and correlations of indicators

Before performing the factor analysis, we reviewed preconditions to begin appropriately and obtain details to further interpret the results. We first prepared our data by eliminating eight outliers to mitigate incorrect results, so our final data set contained 66 user stories. In addition, we evaluated the sample size, which should be between 100 and 200, to obtain more accurate parameters [3]. As our data set is smaller than 100, some limitations may be present in the estimates. An overview of the indicator distributions and correlations is presented in Fig. 2. The results reveal a balanced distribution of semantical quality, a slight left shift distribution of lexical and saturation quality, and a slight right shift distribution of formal quality. Due to the small deviations, we can assume an acceptable normal distribution for all indicators. In addition, we analyze the multicollinearity of the indicators, as high correlations greater than 0.850 can cause problems in estimating parameters [3]. The indicator correlations are low, so we were unable to find multicollinearity.

Next, we tested the overall fit of the indicator specification for user story quality. The analysis resulted in a chi-square ($\chi ^2$) statistic of 4.220 with two degrees of freedom. The p-value was 0.121 at a significance level of $p < 0.050$. This result demonstrates a sufficient overall fit of the model, with a p-value higher than the significance level. In detail, our model can represent the structure in our data set, as the theoretical model-implied indicator correlations are similar to the empirical correlations (see Fig. 2).

In addition, we evaluated the indicator loadings and reliabilities to obtain details regarding the internal consistency of our model. Recommendations of the test theory [3] define indicator loadings higher than 0.300, reliabilities higher than 0.600, and overall reliability with Cronbach’s alpha between 0.800 and 0.900 to be a good fit. Table 1 displays our findings. Semantical quality achieves the best fit, with high loading and reliability. Lexical quality has a medium fit with a moderate loading and low reliability, and both formal quality and saturation quality have a low fit, as they fall below the loading and reliability index. The low agreement with the internal consistency is also confirmed by the global test, as the indicators measure user story quality with a correctness of 0.350, or $35\%$.

**Table 1.** Descriptive statistics and loadings of indicators

Indicator	Mean	Std. Dev.$^\text {a}$	Ind. Loading$^\text {b}$	Alpha ($\alpha$)$^\text {c}$	Comp. Alpha ($\alpha$)$^\text {d}$
Formal quality	4.167	1.223	0.203	0.041	0.350
Lexical quality	42.280	12.345	−0.342	0.117	
Semantical quality	11.347	6.397	0.997	0.995	
Saturation quality	5.258	3.479	−0.106	0.011	

$^\text {a}$ Standard Deviation, $^\text {b}$ Indicator Loading, $^\text {c}$ Cronbach’s Alpha, $^\text {d}$ Composite Cronbach’s Alpha.

In summary, the overall fit suggests that our approach is promising in measuring user story quality with the formal, lexical, semantical, and saturation dimensions. Our indicators subsequently allow for a first evaluation of user story quality in interaction with other constructs (see Fig. 1). However, our instrument is not error free, as indicated by the different loadings and low reliabilities. The first cause for the weakness may be the low sample size and differences in the indicator variances. We will first verify these causes and then enhance the loadings and reliabilities to achieve better evaluation of user story quality in the further ex-post analysis steps. These optimizations helps obtaining details regarding the relationship between quality and human, and project factors, which assists agile requirements engineering research. Deeper insights also have benefits in practice. The identification of “good” criteria aids to prepare design recommendations and AI tools that support authors to write “good” stories.

## References

[1] Beck, K., et al.: The agile manifesto (2001). http://www.agilemanifesto.org. Accessed 13 July 2017

[2] Boehm Barry, Clark Bradford, Horowitz Ellis, Westland Chris, Madachy Ray, Selby Richard (1995). Cost models for future software life cycle processes: COCOMO 2.0. Annals of Software Engineering.

[3] Bühner, M.: Einführung in die Test- und Fragebogenkonstruktion. Pearson, München [u.a.] (2011)

[4] Cohn M (2004). User Stories Applied: For Agile Software Development.

[5] Converse, S., Cannon-Bowers, J. A., Salas, E.: Shared Mental Models in Expert Team Decision Making. Individ. and Gr. Decis. Mak.: Curr. Issues. 221–246 (1993)

[6] Flesch R (1948). A new readability Yardstick. J. Appl. Psychol..

[7] Hallmann, D., Schmid, U., Weth, von der, R.: Gemeinsame mentale Modelle in der agilen Softwareentwicklung: Ein Ansatz zur Erstellung von Gestaltungsempfehlungen für gute“ erfahrungsspezifische User Stories. In: Informatik 2016. LNI, vol. P-259, pp. 1969–1974. GI (2016)

[8] Hallmann, D.: The COCOMO-models in the light of the agile software development. Technical report no. 104/2018, Bamberger Beiträge zur Wirtschaftsinformatik und Angewandeten Informatik, University of Bamberg (2018). 10.20378/irbo-53211

[9] Haugen, N.C.: An empirical study of using planning poker for user story estimation. In: AGILE 2006, pp. 9–34. IEEE (2006). 10.1109/AGILE.2006.16

[10] Jeffries, R.: Essential XP: Card, Conversation, Confirmation. https://ronjeffries.com/xprog/articles/expcardconversationconfirmation/. Accessed 7 Mar 2019

[11] Kline RB (2015). Principles and Practice of Structural Equation Modeling.

[12] Mahnic V, Hovelja T (2012). On using planning poker for estimating user stories. J. Syst. Softw..

[13] Mohammed S, Klimoski R, Rentsch JR (2000). The measurement of team mental models: we have no shared schema. Organ. Res. Methods..

[14] Motogna M, Vaduva S, Fotea IS, Thomas AR (2017). Customer satisfaction in IT professional services research. Development, Growth and Finance of Organizations from an Eastern European Context.

[15] Raith, F., Richter, I., Lindermeier, R., Klinker, G.: Identification of inaccurate effort estimates in agile software development. In: APSEC 2013, pp. 67–72. IEEE (2013). 10.1109/APSEC.2013.114

[16] Ramasubbu, N., Balan, R. K.: Overcoming the challenges in cost estimation for distributed software projects. In: ICSE 2012, pp. 91–101. IEEE (2012). 10.1109/ICSE.2012.6227203

[17] Rodrigo, M. T., Baker, R. S.: Coarse-grained detection of student frustration in an introductory programming course. In: ICER 2009, pp. 75–79. ACM (2009). 10.1145/1584322.1584332

[18] Wake, B.: INVEST in Good Stories, and SMART Tasks. http://xp123.com/articles/invest-in-good-stories-and-smart-tasks/. Accessed 30 Nov 2012

[19] Zelazny, L. M., Belanger, F., Tegarden, D.: Toward a model of information system development success: perceptions of information systems development team members. In: ICIS 2012, pp. 1649–1669. AIS (2012)

